# Micronized Oat Husk: Particle Size Distribution, Phenolic Acid Profile and Antioxidant Properties

**DOI:** 10.3390/ma14185443

**Published:** 2021-09-20

**Authors:** Dariusz Dziki, Wojciech Tarasiuk, Urszula Gawlik-Dziki

**Affiliations:** 1 Department of Thermal Technology and Food Process Engineering, University of Life Sciences in Lublin, Głęboka 31, 20-612 Lublin, Poland; 2 FIBRECARE Sp. z o.o., Słowackiego 16, 40-094 Katowice, Poland; w.tarasiuk@pb.edu.pl (W.T.); urszula.gawlik@up.lublin.pl (U.G.-D.); 3 Faculty of Mechanical Engineering Bialystok, Bialystok University of Technology, Wiejska 45A, 15-351 Białystok, Poland; 4 Department of Biochemistry and Food Chemistry, University of Life Sciences in Lublin, Skromna 8, 20-704 Lublin, Poland

**Keywords:** oat hull, micronization, impact classifier mill, microbiological purity, particle size distribution, total phenolic content, antioxidant properties

## Abstract

Oat husk (OH; hull) is a by-product generated from oat processing and is rich in insoluble fibre and phenolic compounds. The aim of this work was to study the particle size distribution, antioxidant activity, and phenolic profile of micronized OH. For this purpose, the hull was first sterilized using superheated steam and was then ground using an impact classifier mill. The particle size distribution (PSD) of the ground husk was determined using the laser diffraction method and the parameters characterizing the PSD of the ground husk, and its antioxidant activity were calculated. In addition, UPLC-MS/MS analysis of phenolic acids was also performed. Micronization of the sterilized husk effectively decreased the size of the particles, and with the increasing speed of the rotor and classifier, the median size of the particles (d_50_) decreased from 63.8 to 16.7 µm. The following phenolic acids were identified in OH: ferulic, caffeic, p-hydroxybenzoic, vanillic, syringic, and synapic acid. Ferulic acid constituted about 95% of total phenolic acids. The antioxidant activity of the obtained extracts increased as the particle size of the micronized husk decreased. The highest half maximal inhibitory concentration (EC_50_ index) was found for chelating power, and the lowest was found in the case of radical scavenging activity against DPPH.

## 1. Introduction

Oat is becoming more popular as a raw material in various sectors of the food industry because of the well-known health benefits of its products. Oat grain is rich in many bioactive compounds such as phenolic acids, flavonoids, avenanthramides, and fibre [1,2]. Oat husk (OH; hull) is a by-product obtained from oat processing for food purposes. It is assumed that 2.75–3.3 million tons of OH is generated as a by-product [3]. OH usually represents an average of 27% of oat grain [4]. Emmons and Peterson [5] found a similar level of total phenolics in OH as in oat groat and husked oat. However, studies conducted by Piątkowska et al. [6] on different parts (e.g., husk, bran, endosperm and whole grain) of 13 oat varieties showed that OH contained the highest level of polyphenols and exhibited the highest antioxidant activity. Additionally, OH is a rich source of insoluble dietary fibre with health-promoting properties. This type of fibre includes cellulose and lignins, which are the components of plant cell walls, and absorbs water but does not dissolve in it. Dietary fibre increases the volume and softness of the stool and supports normal intestinal peristalsis [7]. The main constituent of OH is lignocellulosic fibres, which are made up of cellulose, hemicelluloses, and lignin [8]. The composition of OH is as follows: ~33% crude fibre, ~33% pentosans, ~13% lignin, ~4% protein, and ~5% ash [9]. OH is mainly used in animal feed or in the production of industrial solvents. However, it can also be used as an ingredient in high-dietary fibre foods [10]. Studies analysing the possibility of using OH as a food additive are limited in the literature. Piwińska et al. [11] added a mixture of OH with a soluble fraction of oat and produced wheat pasta. Oliveira et al. [8] showed that OH is a valuable source of cellulose fibres that can be used in the production of hydrogels that can be used in different applications.

Grinding is an important process that is followed in the food industry. The particle size of the ground seeds determines the properties of the final products [12,13]. Ultra-fine grinding (micronization), in particular, has become very popular in recent years [14,15,16]. Micronization is a process by which the particle size of a material is reduced to a nanosize range [17,18], thereby improving dispersion, chemical and biological activities, and the rate of nutritional absorption from food [19]. In fibre-rich plant materials, micronization enhances the water absorption of the particles and the solubility of fibre [20]. It also improves mouthfeel and increases the release of flavours [18]. Importantly, micronization improves the extraction of antioxidant compounds and helps in the release of many bioactive compounds bound to the food matrix [19,21,22].

Due to its high fibre content, the proper grinding of OH using traditional size reduction methods is difficult. Moreover, after conventional grinding, the OH particles produce an unpleasant, prickly mouth feeling [23]. Therefore, the fine grinding of such a material is recommended. Currently, no studies on the process of OH micronization and its properties can be found in the literature. Therefore, this work aimed to study the influence of the working parameters of an impact classifier mill on the particle size distribution (PSD), phenolic profile, and antioxidant properties of pulverized OH.

## 2. Materials and Methods

OH was received from AG Feeding Sp. z o.o. (Gdynia, Poland). Folin–Ciocalteu reagent, DPPH (2,2–diphenyl-1-picrylhydrazyl), gallic acid monohydrate, methanol, and ABTS (2,2-diphenyl-1-picrylhydrazyl) were purchased from the Sigma–Aldrich company (Poznań, Poland). Methanol and acetonitrile HPLC grade were purchased from Merck (Darmstadt, Germany). Formic acid LC-MS grade was purchased from Merck (Darmstadt, Germany). Ultrapure water was obtained in-house with a purification system (Milli-Q-Simplicity-185, Millipore Corp., Burlington, MA, USA). Reference standard kaempferol was purchased from Fluka AG (Buchs, Switzerland). All chemicals were of analytical grade.

### 2.1. Basic Composition

The moisture content (Method 925.10), crude protein content (Method 992.33; Nx6.25), ash content (Method 942.05), and total fibre content (Method 985.29) of OH were determined using standard methods [AOAC, 2011] [24].

### 2.2. Husk Sterilization

Before being ground, OH was sterilized using superheated steam generated from a thermal sterilizer of the authors’ own design. The sterilizer consisted of an insulated heated chamber equipped with a paddle mixer and a superheated steam supply system. The screw conveyor delivered the material to be ground to the heated chamber, the walls of which were heated up to a temperature of 200 °C. The paddles in the sterilizer chamber moved the material while mixing it. This prevented the material that came in direct contact with the chamber wall for a long time from burning. The raw supplied material was heated by the walls of the sterilizer. After preheating the material, a superheated steam that was 200 °C was supplied to the chamber, which had a total length of 6 m. The exhaust hood used at the end of the chamber removed the excess steam and did not allow the steam to retract. The total sterilization time was 4 min. The material from the sterilizer went directly into the cooling conveyor, which also had a length of 6 m. The efficiency of the sterilization and cooling process was identical;therefore, the material that was cooled to a temperature of about 30 °C was sent directly to the mill.

### 2.3. Microbiological Purity of OH

The OH samples were analysed before and after sterilization for the total number of yeasts and moulds [25], the total number of microorganisms [26], coliform counts [27], and *Salmonella* [28] and *Bacillus cereus* counts [29].

### 2.4. Micronization of OH

The sterilized OH was micronized using an impact classifier mill equipped with a rotor disc with 12 hammers and a classifier [30]. The mill was integrated with a centrifugal fan (airflow 2 m^3^∙s^−1^). The OH was ground by setting the disc at four different speeds: 2600, 2970, 3340, and 3710 rpm. It was pulverized by the hammers and passed through a liner with a corrugated surface. The pulverized material was then transported through a baffle assembly into a classifier wheel, which restricts the coarse particles from escaping to outside the mill, and these particles were recirculated back into the milling chamber. For each speed of the rotor disc, six different classifier wheel speeds were applied: 480, 965, 1450, 1930, 2410, and 2890 rpm. A detailed description of the mill used in this study was given by Dziki et al. [30].

### 2.5. Particle Size Distribution 

The PSD of the micronized OH was measured by laser light scattering from 0.01 to 3.0 mm using a laser particle size analyser (Malvern Mastersizer 3000, Malvern Instruments Ltd., Worcestershire, UK). A total of 5 g of micronized OH sample was added to the inlet chamber, and the PSD was measured automatically by laser diffraction using the dry dispersion method [31]. PSD was expressed as d_10_, d_50_ (median diameter), and d_90_, which represent the 10th, 50th, and 90th percentile of the total volume, assuming the particles have a spherical shape. Moreover, the volume-based size distribution (*Span*) was calculated [32] using the following equation:

(1) $$Span=\frac{d_{90}-d_{10}}{d_{50}}$$

### 2.6. Antioxidant Properties

To study the antioxidant activity, 50% methanolic (methanol: water, 1:1 *v*/*v*) extracts of micronized OH were prepared. An amount of 250 mg of plant material was triple extracted using 5 mL of 50% aqueous methanol in a laboratory shaker for 30 min and then centrifuged. The combined extracts were used for further analyses [33].

The antioxidant activity of the OH extracts was determined by three assays: chelating power (CHEL), DPPH, and the ABTS radical scavenging activity. CHEL was determined according to the method presented by Guo et al. [34] with slight modification [35]. Radical scavenging activity of the OH extract against stable DPPH and the ABTS radical scavenging activity were determined according to Brand-Williams et al. [36] and Re et al. [37], respectively. The blank samples contained 50% methanol instead of the extract.

The chelating potential was assessed according to the formula:

% inhibition = [1 − (AA/AC)] × 100(2)

Free radical scavenging activity was assessed according to the following equation:

scavenging % = [(AC − AA)/AC)] × 100(3)

where AC is the absorbance of the control, and AA is the absorbance of the sample.

The half maximal inhibitory concentration (EC_50_) values were calculated at fitted models, as the concentration of the tested compound produced 50% of the maximum inhibition based on a dose-dependent mode of action [38].

### 2.7. UPLC-MS/MS Phenolics Acids Analysis

Three OH samples (200 mg each) of OH with the highest degree of fineness were selected and hydrolyzed with 4N NaOH and 2% ascorbic acid solution for 4 h at room temperature. Then, the samples were cooled in ice and were acidified using ice-cold 6M HCl to achieve a pH value of about 2. The resulting mixtures were centrifuged at 8000 rpm (Sigma 2-16) for 20 min. Supernatants of ethyl acetate were extracted three times. The organic phase was collected, filtered, and evaporated to dryness at 35 °C in a rotary evaporator. The residue was dissolved in 30% methanol and was stored in a freezer.

The phenolic acids were determined by an ACQUITY UPLC system equipped with a PDA and a triple quadrupole mass detector (TQD, Waters, Milford, MA, USA). Samples were separated on a Waters Acquity UPLC HSS C18 column (100 × 2.1 mm, 1.8 µm) with a mobile phase of solvent A (acidified water, 0,1% formic acid) and solvent B (acidified acetonitrile, 0,1% formic acid). The solvent gradient was programmed as follows: 8% to 20% B in 8 min; 20% to 95% B in 2.9 min; and 95% to 8% B in 2 min at a flow rate of 0.5 mL/min. The column temperature was maintained at 30 °C. The injection volume was set to 2.5 μL. Compounds were interpreted based on data from mass spectra. ESI ionization was performed in negative ion mode. Data processing was performed using MassLynx V4.1 software, Waters (Milford, MA, USA).

### 2.8. Statistical Analysis

All measurements were performed in triplicate unless otherwise noted. Analysis of variance was performed (one-way or two-way), and significant differences between means were determined using Tukey’s test. In addition, correlation and multilinear regression analyses were performed. The significance level of α was established at 0.05. Statistica 13.3 software (StatSoft, Tulsa, OK, USA) was used for the calculations.

## 3. Results and Discussion

### 3.1. Basic Composition and Microorganisms Identification

The basic chemical composition of OH was as follows: ash content 3.42 ± 0.11%, protein content 1.27 ± 0.08%, total fibre content 90.7 ± 1.23%, and initial moisture content 10.2 ± 0.15%. The microorganisms identified in the OH before and after sterilization are summarized in Table 1. Microorganisms are an integral part of cereal grains. The external parts of grains such as OH are especially susceptible to contamination during the period of grain ripening, harvesting, and storage [39]. Although microorganisms do not grow under lower water activity, bacteria and fungi can thrive for a long time, and under favourable conditions, they become active and present a potential hazard [40]. *Escherichia coli*, *B. cereus,* and *Salmonella* were not detected both before and after sterilization in any of the OH samples. However, moulds and yeasts were detected. The average number of moulds and yeasts and the total number of bacteria before sterilization were 4.0·10^5^ and 4.6·10^6^ cfu·g^−1^, respectively. Sterilization significantly decreased the number of microorganisms to an acceptable level [41]. The numbers of yeasts and moulds were found to have been decreased below 10^1^ cfu·g^−1^, whereas the average total aerobic plate count was 2.6·10^3^ cfu·g^−1^. According to the literature data, the content of yeasts and moulds in healthy cereal grains and oilseeds does not exceed 10^4^ cfu·g^−1^ [42]. In addition, in the study, it was observed that sterilization decreased the moisture of the hull by up to 3.4 ± 0.22%. A decrease in the moisture content of the plant materials is known to increase their friability and usually results in the grinding process being more effective [43,44].

### 3.2. Particle Size Distribution

**Figure 1:**
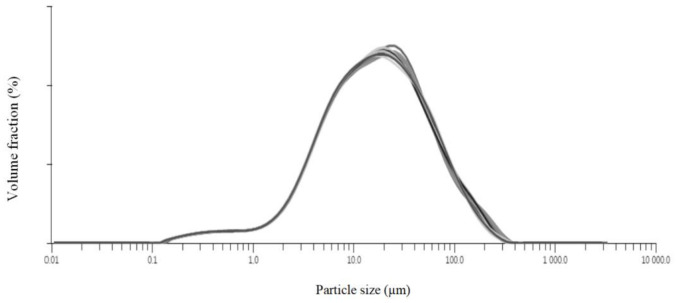
Example curves of the PSD of OH (sample H18, eight repetitions).

Example curves of the PSD of OH are presented in Figure 1, and the parameters that describe the PSD of ground OH are summarized in Table 2. The values of d_10_ changed in a relatively narrow range from 3.2 to 7.8 µm, whereas those of d_50_ and d_90_ ranged from 16.7 to 63.8 µm and from 75.1 to 282 µm, respectively. In the case of *Span*, which refers to the PSD width, the values increased from 4.1 to 7.9. The degree of fineness of the fibre-rich oat flour has a significant influence on the properties of many final products, such as bread [45], pasta [46], and sausage [47]. Particle size affects the texture and mouthfeel [48] of products and increases their bioavailability [49].

In general, the higher the speed of both the disc and classifier, the lower the values of d_10_, d_50_, and d_90_. The results of the variance analysis of the parameters that characterize the particle size of the micronized OH are presented in Table 3. The speed of rotation of both the disc (*v_d_*) and classifier (*v_k_*) had a significant influence on the parameters characterizing the PSD of the OH. However, according to the values obtained by the *F*-test, the speed of the classifier had the highest influence on d_10_, d_50_, and d_90_, whereas the speed of the rotor disc had a crucial effect on the parameter *Span*.

Moreover, in the case of all of the parameters, significant interactions were found between the speed of the disc and the classifier. The changes in these parameters were described using multiple linear regression:

(4) $$Y=a_{1}\times v_{d}+a_{2}\times v_{k}+b\;\pm e$$

where *Y* is the dependent variable, *a*_1_ and *a*_2_ are the regression coefficients, *b* is the intercept, *v_d_* and *v_k_* are the speeds of the disc and classifier, respectively, and *e* is the model’s error.

The results of the regression are summarized in Table 4. All of the parameters describing the PSD showed significant relations, and the coefficient of determination ranged from 0.722 (d_10_) to 0.785 (d_50_). This shows that the proposed equations generally described the changes in d_10_, d_50_, and d_90_ well with respect to the function of the rotation speed of the disc and the classifier. In the case of *Span*, the lowest *R*^2^ values were found (0.577). However, for both the coefficient of determination and the equation parameters, *p*-values were statistically significant.

### 3.3. Antioxidant Activity (AA) of OH

OH is rich in phenolic acids such as ferulic acid, *p*-coumaric acid, and vanillic acid, which have an influence on its AA [50]. Vagra et al. [51] studied the phenolic composition and AA of oat hull and groats using 20 oat genotypes and found AA that was several times higher in husks compared to groats. Lower values of d_10_, d_50_, and d_90_ resulted in a lower EC_50_ index in the case of all AA assays and consequently higher antioxidant activity (Table 5). The values of EC_50_ for CHEL changed from 27.67 mg dm/mL (H24) to 37.05 mg dm/mL (H1). The values of EC_50_ for ABTS ranged from 35.3 mg dm/mL (H23) to 44.9 mg dm/mL (H6), and the values for DPPH ranged from 76.9 mg dm/mL (H11) to 85.1 mg dm/mL (H7).

The coefficients of correlation between d_10_, d_50_ d_90_ and CHEL were statistically significant and were included in Table 6. A similar relation was found between the particle size of micronized wheat bran and antioxidant activity in the study by Rosa et al. [52], in which a 1.5-fold increase was observed in the antioxidant capacity of bran when d_50_ was decreased from 172 to 30 µm. Additionally, other authors have observed that antioxidant activity increased as a result of the micronization of different fibre-rich plant materials [53,54,55]. This fact can be explained by the expansion of the surface area of particles and the destruction of the dietary fibre matrix, which resulted in the release of some antioxidative compounds from the matrix. Thus, the ultra-fine grinding of fibre-rich plant materials could be beneficial in enhancing the antioxidant activity and the pro-health value of food. In addition, the reduction of particle size by micronization increases the bioavailability of many compounds, and pulverized material is easily absorbed by the human body [18]. The relatively lower values of the antioxidant activity of OH can result from the interactions of polyphenols with carbohydrates originating from the cell wall such as cellulose or dietary fibres [56]. However, the polyphenols from such complexes could be released in the colon when exposed to various enzymes and microorganisms [57,58,59,60].

### 3.4. Phenolic Acids Profile

The UPLC-MS/MS analysis detected seven phenolic acids in the OH samples. The three samples of OH (H12, H17 and H24) with the highest degree of fineness (the lowest values of d_50_) were not statically different according to phenolic acid profile. The dominant phenolic acid was ferulic acid (on average 454.56 µg/mg d.m.). This compound represented about 95% of all of the detected phenolic acids. The following phenolic acids were detected in the OH samples: caffeic, *p*-hydroxybenzoic, vanilic, syringic, and synapic acid (Table 7). This compound has a lot of applications. It is an ingredient in many drugs, functional foods, and nutraceuticals [61]. Ferulic acid (4-hydroxy-3-methoxycinnamic acid) shows anticancer, anti-inflammatory, antimicrobial, antithrombotic, and hepatoprotective activity [62]. Although ferulic acid is the main phenolic acid of OH, it is not effortlessly available from this material because it is covalently linked with lignin and other biopolymers [62]. However, the enzymatic production of ferulic acid from lignocellulosic materials is possible [63,64].

## 4. Conclusions

This study demonstrated that the sterilization of OH decreases the moisture content, thereby resulting in a very dry material with appropriate microbiological purity. The proposed method of micronization effectively reduced the particle size of the sterilized husk. In addition, PSD can be modified by changing the working parameters of the mill that is used. By increasing the speed of hammers and classifier, ground OH with a median particle size below 20 µm can be obtained. Moreover, the association between the working parameters of the mill and the parameters describing the PSD of OH can be described by using a multilinear equation with respect to the speeds of both the disc and the classifier. Most importantly, the obtained material is rich in phenolic compounds, and a positive correlation was found between the size of the particles and the antioxidant activity. The UPLC-MS/MS analysis showed that ferulic acid represented about 95% of all of the detected phenolic acids. Therefore, the powder obtained from the OH can be used as a functional food additive and as a source of ferulic acid for industrial purposes.

## Figures and Tables

**Table 1:** Microorganisms (cfu·g^−1^) identified in the OH before and after sterilization.

Oat Husk	Yeast and Moulds	Aerobic Plate Count	*Escherichia coli*	*Batocillus Cereus*	*Salmonella*
Before Sterilization	4.0·10^5^ ± 2.0·10^5^	4.6·10^6^ ± 2.3·10^6^	nd *	nd	nd
After Sterilization	<10^1^	2.6·10^3^ ± 0.5·10^1^	nd	nd	nd

* not detected.

**Table 2:** Parameters that describe the particle size distribution of micronized OH.

Sample	Revolution of Disc (rpm)	Revolution of Classifier Wheel (rpm)	d_10_ (µm)	d_50_ (µm)	d_90_ (µm)	Span
H1	2600	480	6.2 ± 0.05 ^m,^*	41.3 ± 1.02 ^h^	249 ± 11.4 ^j,k^	5.9 ± 0.16 ^i^
H2	2600	965	6.1 ± 0.07 ^m^	55.7 ± 3.76 ^j^	282 ± 16.0 ^n^	5.0 ± 0.23 ^g^
H3	2600	1450	5.5 ± 0.05 ^l^	50.6 ± 1.33 ^i^	277 ± 5.1 ^m,n^	5.4 ± 0.12 ^h^
H4	2600	1930	5.3 ± 0.04 ^k^	47.6 ± 1.45 ^i^	269 ± 6.9l ^m,n^	5.5 ± 0.04 ^h^
H5	2600	2410	4.9 ± 0.04 ^i^	40.0 ± 0.71 ^h^	264 ± 4.6l ^m^	6.5 ± 0.11 ^j^
H6	2600	2890	4.2 ± 0.02 ^e^	22.9 ± 0.24 ^d,e^	185 ± 3.1 ^f^	7.9 ± 0.11 ^l^
H7	2970	480	7.1 ± 0.09 ^n^	56.1 ± 3.18 ^j^	268 ± 10.9 ^l,m^	4.6 ± 0.10 ^e,f^
H8	2970	965	5.1 ± 0.07 ^k^	42.6 ± 1.50 ^h^	233 ± 3.5 ^h,i^	5.4 ± 0.11 ^h^
H9	2970	1450	4.7 ± 0.07 ^h^	40.1 ± 3.97 ^h^	224 ± 17.1 ^h^	5.5 ± 0.13 ^h^
H10	2970	1930	3.8 ± 0.04 ^d^	26.3 ± 0.78 ^f^	179 ± 7.3 ^f^	6.7 ± 0.10 ^j^
H11	2970	2410	3.8 ± 0.07 ^d^	20.8 ± 0.95 ^c,d^	150 ± 7.2 ^d^	7.1 ± 0.23 ^k^
H12	2970	2890	3.4 ± 0.03 ^c^	15.5 ± 0.22 ^a^	105 ± 4.3 ^b^	6.6 ± 0.2^1 j^
H13	3340	480	7.7 ± 0.04 ^o^	63.8 ± 1.67 ^k^	261 ± 4.7 ^k^	4.0 ± 0.06 ^a,b^
H14	3340	965	4.1 ± 0.23 ^e^	25.8 ± 3.98 ^e,f^	174 ± 19.9 ^e,f,l^	6.6 ± 0.28 ^j^
H15	3340	1450	5.0 ± 0.07 ^j^	41.2 ± 0.89 ^h^	203 ± 4.7 ^g^	4.8 ± 0.07 ^f,g^
H16	3340	1930	4.3 ± 0.08 ^f^	28.8 ± 0.81 ^f^	180 ± 8.0 ^f^	6.1 ± 0.15 ^i^
H17	3340	2410	3.4 ± 0.05 ^b,c^	20.2 ± 0.41b ^c,d^	125 ± 2.6 ^c^	6.0 ± 0.11 ^i^
H18	3340	2890	3.2 ± 0.03 ^a^	17.4 ± 0.16 ^a,b^	99 ± 2.3 ^b^	5.5 ± 0.11 ^h^
H19	3710	480	7.8 ± 0.12 ^o^	67.4 ± 1.26 ^l^	265 ± 5.1 ^l,m^	3.8 ± 0.11 ^a^
H20	3710	965	7.1 ± 0.09 ^n^	57.1 ± 1.04 ^j^	243 ± 9.0 ^I,j^	4.1 ± 0.11 ^b,c,d^
H21	3710	1450	4.5 ± 0.05 ^g^	34.5 ± 0.66 ^g^	161 ± 3.9 ^d^	4.5 ± 0.09 ^e^
H22	3710	1930	4.2 ± 0.04 ^e^	26.2 ± 0.32 ^f^	113 ± 1.8 ^b,c^	4.2 ± 0.07 ^b,c^
H23	3710	2410	3.3 ± 0.03 ^c,b^	18.2 ± 0.41 ^b,c^	78 ± 1.8 ^a^	4.1 ± 0.17 ^b^
H24	3710	2890	3.3 ± 0.05 ^b,c^	16.7 ± 0.29 ^a^	75.1 ± 1.8 ^a^	4.3 ± 0.12 ^c,d^

* The values designated by the different small letters (a, b, c, d, …) are significantly different (α = 0.05).

**Table 3:** The analysis of variance for the parameters characterizing the PSD of pulverized oat husk.

Parameter	Source of Variance	Sum of Squares	Degrees of Freedom	Mean Square	*F*-Test	*p*-Value
d_10_	v_d_ *	5.34	3	1.78	283	0.0001
	*v_k_*	27.06	5	5.41	859	0.0001
	*v_d_·v_k_*	17.43	15	1.16	184	0.0001
	Error	0.756	120	0.006	-	-
d_50_	*v_d_*	943.5	3	314.5	692.4	0.0001
	*v_k_*	4976.3	5	995.3	2191.3	0.0001
	*v_d_·v_k_*	3592.5	15	239.5	527.3	0.0001
	Error	54.5	120	0.5	-	-
d_90_	v_d_ *	23.225	3	7742	647.4	0.0001
	*v_k_*	147.444	5	29.489	2466	0.0001
	*v_d_·v_k_*	94.26	15	6284	525.5	0.0001
	Error	1435	120	12	-	-
*Span*	v_d_ *	21.1	3	7.04	813.6	0.0001
	*v_k_*	11.3	5	2.26	260.5	0.0001
	*v_d_·v_k_*	27.4	15	1.83	211.6	0.0001
	Error	1.04	120	0.009	-	-

* *v_d_*—speed of the disc, *v_k_*—speed of the classifier.

**Table 4:** Results of equations describing the relationships between the working parameters of the mill and the parameters describing the PSD of micronized OH.

Dependent Variable	Equation Parameter	Standardized Coefficient of Regression	Standard Error	Coefficient of Regression	Standard Error	t (213)	*p*-Value	*R* ^2^	Model’s Error
d_10_	Intercept	-	-	8.155	0.402	20.262	0.00001	0.722	0.740
	v_d_ *	−0.846	0.0361	−0.00144	0.00006	−23.420	0.00001		
	*v_k_*	−0.077	0.0361	−0.00026	0.00012	−2.118	0.03534		
d_50_	Intercept	-	-	79.94267	4.76189	16.788	0.00001	0.785	8.79
	*v_d_*	−0.822	0.038	−0.01579	0.00073	−21.718	0.00001		
	*v_k_*	−0.139	0.038	−0.00532	0.00145	−3.677	0.00001		
d_90_	Intercept	-	-	561.98	17.08	32.91	0.00001	0.784	31.5
	*v_d_*	−0.716	0.03178	−0.0587	0.00261	−22.52	0.00001		
	*v_k_*	−0.522	0.03178	−0.0852	0.00519	−16.42	0.00001		
Span	Intercept	-	-	9.5329	0.38747	24.60	0.00001	0.577	0.715
	*v_d_*	−0.6143	0.0445	−0.0016	0.00012	−13.79	0.00001		
	*v_k_*	0.4471	0.0445	0.0006	0.00006	10.02	0.00001		

* *v_d_*—speed of the disc, *v_k_*—speed of the classifier.

**Table 5:** Antioxidant activity of OH.

Sample	EC_50CHEL_ [mg dm/mL]	EC_50ABTS_ [mg dm/mL]	EC_50DPPH_ [mg/mL]
H1	37.05 ± 1.35 ^h,i^	41.3 ± 1.2 ^a–g^	80.5 ± 1.2 ^a,b,c^
H2	37.79 ± 0.40 ^i^	42.2 ± 2.1 ^c–g^	82.5 ± 1.92 ^a,b,c^
H3	36.23 ± 0.36 ^h,i^	43.2 ± 2.1 ^e,f,g^	80.9 ± 1.42 ^a,b,c^
H4	36.40 ± 0.41 ^h,i^	44.9 ± 1.8 ^g^	82.2 ± 2.42 ^a,b,c^
H5	35.27 ± 0.34 ^f–i^	44.1 ± 1.4 ^f,g^	82.1 ± 1.72 ^a,b,c^
H6	28.20 ± 0.36 ^a,b^	41.9 ± 1.6 ^b–g^	77.8 ± 1.72 ^a,b,c^
H7	36.30 ± 0.45 ^h,i^	44.4 ± 2.0 ^g^	85.1 ± 1.4 ^a,c^
H8	34.47 ± 0.64 ^f,g,h^	43.0 ± 2.0 ^e,f,g^	81.3 ± 1.12 ^a,b,c^
H9	34.83 ± 0.57 ^f,g,h^	40.4 ± 1.3 ^a–g^	82.1 ± 1.72 ^a,b,c^
H10	29.75 ± 0.85 ^a–d^	37.9 ± 2.3 ^a–e^	77.5 ± 1.22 ^a,b,c^
H11	29.73 ± 0.55 ^a,b,c^	38.1 ± 1.6 ^a–f^	76.9 ± 1.3 ^a^
H12	28.06 ± 0.49 ^a,b^	35.9 ± 1.6 ^a,b^	78.7 ± 3.4 ^a,b^
H13	35.64 ± 0.82 ^g,h,i^	42.6 ± 2.6 ^d,e,f,g^	81.8 ± 3.72 ^a,b,c^
H14	30.51 ± 0.36 ^a–e^	41.2 ± 2.7 ^a–g^	79.4 ± 2.72 ^a,b,c^
H15	33.27 ± 1.11 ^e,f,g^	42.9 ± 2. 5 ^e,f,g^	83.2 ± 0.52 ^a,b,c^
H16	32.67 ± 0.82 ^d,e,f^	39.9 ± 2.17 ^a–g^	81.0 ± 3.82 ^a,b,c^
H17	30.6 ± 1.09b ^c,d,e^	36.4 ± 2.00 ^a,b,c^	77.2 ± 1.8 ^a^
H18	28.77 ± 0.79 ^a,b,c^	36.5 ± 0.9 ^a,b,c,d^	78.0 ± 1.2 ^a,b^
H19	36.37 ± 0.91 ^h,i^	41.4 ± 1.67 ^a–g^	83.6 ± 1.02 ^a,b,c^
H20	37.33 ± 1.15 ^h,i^	42.4 ± 2.2 ^c–g^	83.0 ± 2.12 ^a,b,c^
H21	34.60 ± 0.72 ^f,g,h^	37.5 ± 2.1 ^a–e^	80.9 ± 1.72 ^a,b,c^
H22	31.33 ± 1.10 ^c,d,e^	37.2 ± 2.1 ^a–e^	77.1 ± 1.6 ^a^
H23	31.27 ± 0.76 ^c,d,e^	35.3 ± 2.1 ^a^	77.9 ± 2.2 ^a,b^
H24	27.67 ± 2.59 ^a^	35.6 ± 1.6 ^a^	79.7 ± 3.2 ^a,b^

The values designated by the different small letters (a, b, c, d, …) are significantly different (α = 0.05).

**Table 6:** Correlation coefficients and *p*-values describing the relation between the parameters of PSD and antioxidant activity of OH.

Parameter	CHEL *		ABTS		DPPH	
	r	*p*-Value	r	*p*-Value	r	*p*-Value
d_10_	0.822	*p* = 0.0001	0.752	*p* = 0.0001	0.854	*p* = 0.0001
d_50_	0.887	*p* = 0.0001	0.696	*p* = 0.0001	0.795	*p* = 0.0001
d_90_	0.845	*p* = 0.0001	0.908	*p* = 0.0001	0.798	*p* = 0.0001

* CHEL—chelating power, ABTS—radical scavenging activity, DPPH—antiradical activity.

**Table 7:** Total phenolic acids profile (free and bound, µg/mg d.m.) in OH.

Phenolic Acid	Sample		
	H12	H17	H24
Protocatechuic acid	0.73 ± 0.02 ^a^	0.76 ± 0.01 ^a^	0.74 ± 0.05 ^a^
*p*-Hydroxybenzoic	5.12 ± 0.07 ^a^	5.13 ± 0.17 ^a^	5.05 ± 0.16 ^a^
Vanillic	4.26 ± 0.31 ^a^	4.24 ± 0.27 ^a^	4.33 ± 0.12 ^a^
Caffeic	6.26 ± 0.01 ^a^	6.28 ± 0.10 ^a^	6.18 ± 0.21 ^a^
Syringic	3.14 ± 0.17 ^a^	3.07 ± 0.16 ^a^	3.03 ± 0.18 ^a^
*p*-Coumaric	Nq **	Nq	Nq
Ferulic	438.19 ± 14.32 ^a^	427.93 ± 8.18 ^a^	433.37 ± 6.79 ^a^
Synapic	1.84 ± 0.07 ^a^	1.92 ± 0.13 ^a^	1.83 ± 0.11 ^a^
Salycilic	Nd	Nd	Nd
Total	459.55 ± 14.22 ^a^	449.32 ± 8.28 ^a^	454.52 ± 7.22 ^a^

The values designated by the same small letters (a) are not significantly different (α = 0.05). ** Nq—above limit of detection and below limit of quantification, Nd—below limit of detection.

## Data Availability

The data presented in this study are available upon request from the corresponding author.
